# Paths of Suicidal Ideation Identification and Suicidal Behavior Intervention: A Qualitative Comparative Analysis of Chinese Young People

**DOI:** 10.3390/healthcare13233128

**Published:** 2025-12-01

**Authors:** Yaping Xin, Xuanyuan Chen, Dan Li

**Affiliations:** 1 Students Affairs Department, Sichuan University, Chengdu 610065, China; xinya_ping@163.com; 2 School of Public Administration, Sichuan University, Chengdu 610065, China; ldan99@126.com

**Keywords:** suicide identification, suicide intervention, Chinese young people, school support, family support

## Abstract

**Objective:** This study examines pathways for suicide ideation identification and suicidal behavior intervention among Chinese young adults. **Methods:** It used qualitative comparative analysis (QCA) to analyze the cases of 47 Chinese young people (aged 18–28) with suicidal experiences. The outcome variables are suicide ideation identification and suicidal behavior intervention, and condition variables include psychological disorders, suicidal history, suicidal communication, suicidal time, suicidal location, suicidal methods, family support, peer support, and school support. **Results:** There are two successful identification pathways and five effective intervention pathways, contrasted with four failed identification pathways and one failed intervention pattern. These results reveal that continuous supervision of individuals with psychological disorders and multi-group participation in intervention are important to decrease the suicide risk of Chinese young people. Meanwhile, a lack of proactive identification of individuals without warning signals, insufficient attention from families and communities to young people with psychological disorders, and inadequate physical limitations on fatal suicidal behaviors are major risk factors. **Conclusions:** This study highlights the measures of strengthening continuous attention to suicide signals among high-risk youth groups, limiting lethal suicide methods, promoting network monitoring and suicide risk warning, increasing psychological services in the community, and improving the collaborative synergy of peers, families, and schools.

## 1. Introduction

Suicide has become a critical global public health issue. The World Health Organization reports that 726,000 people die by suicide each year, with an estimated 20 attempts for every completed suicide [1]. This alarming trend is particularly severe among young people, as suicide has risen from the fourth to the third leading cause of death among individuals aged 15 to 29 globally between 2019 and 2021 [1,2]. Teenagers have emerged as a vulnerable group with an increased risk of suicide. Addressing the issue of suicide among young adults has become a significant global challenge, especially in China, where suicide deaths account for one-fifth of the world’s total suicides [3]. Additionally, this demographic experiences higher rates of suicidal thoughts and attempts compared to young adults in other countries [4].

Suicide is a conscious act of self-induced annihilation [5] and results from a combination of various multidimensional factors, including individual, family, and societal influences [6]. From an individual perspective, youth are at an increased risk for suicide due to factors such as psychological disorders, a history of suicide attempts, substance abuse, and internet addiction [7,8,9]. At the family level, factors including family conflict, excessive supervision, parental neglect, and low socioeconomic status also contribute to an elevated risk of suicide among youth [10,11,12]. Additionally, social factors such as bullying on campus, neighborhood violence, adverse cultural influences, and inadequate social policies are significant contributors to youth suicide [13,14,15,16]. These findings provide substantial theoretical support for interventions aimed at preventing youth suicide.

With the development of interpersonal suicide theory and the three-step theory, scholars pay more attention to the suicide process. Studies have demonstrated that pain, hopelessness, and the combination of the two are determinants of suicidal ideation, and social disconnectedness leads to an escalation of suicidal desires, evolving into suicidal planning and actions [17,18,19,20]. Suicidal capability is crucial for distinguishing between those who contemplate suicide and those who attempt it. Factors like pain tolerance, fearlessness regarding death, and practical capability are linked to a higher likelihood of engaging in both suicide attempts and actions [21,22,23]. Furthermore, individuals who complete suicide tend to use more lethal methods, such as hanging, shooting, or jumping from heights, rather than less lethal means like drug poisoning or stabbing [24,25,26]. However, there is limited research examining the differences between attempted and completed suicide from the perspective of intervention throughout the suicide process.

Many effective intervention measures have been proposed in existing research, such as prevention plans, psycho-education courses, mental health screening, gatekeepers, psychological counseling, restricting access to lethal means, and a peer-to-peer approach to suicide prevention [27,28,29,30,31]. However, there are very few empirical studies that specifically focus on intervening in the youth suicide process. Therefore, this study aims to explore the differences between suicide attempters and suicide completers to identify suicidal ideation and intervene in suicidal behaviors among young people.

Suicide involves an ideation-to-action complex process, including suicidal ideation, suicidal communication, suicide attempts, suicidal planning, and completed suicide [15,32,33]. According to the three-step theory, there are two critical points for intervention. The first is to limit cognitive access to reduce the risk of suicidal thoughts escalating into attempts. The second is to restrict physical conditions that could lead to attempts to complete suicide [17,34]. These findings provide a theoretical framework for this study.

**Figure 1:**
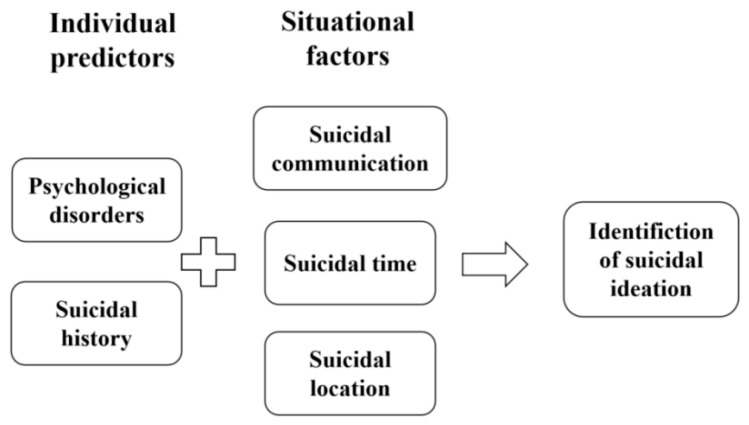
A conditional combination model for the paths of identifying suicidal ideation among youth groups.

Research has shown that psychological disorders and a history of suicide attempts are regarded as the most effective predictors of suicidal behaviors [7,8,35]. From the perspective of situational factors, studies have indicated that suicidal communication provides a key window of opportunity for gatekeepers to prevent suicide [36,37,38]. Furthermore, suicidal behaviors that occur in public spaces or during the daytime are more easily recognized [39,40]. Therefore, this study selects psychological disorders and a history of suicide attempts as individual predictors, while focusing on suicidal communication, the timing of suicidal actions, and the locations of suicidal behaviors as situational factors in identifying suicidal ideation. A conditional combination model illustrating the pathways for identifying suicidal ideation among youth is presented in Figure 1.

**Figure 2:**
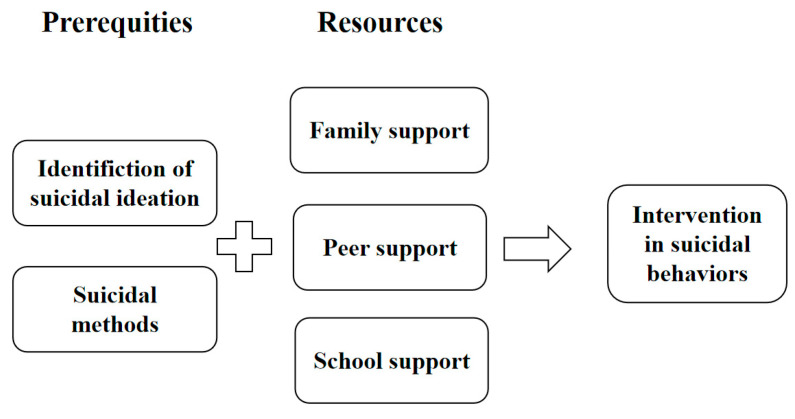
A conditional combination model for the paths of intervening in suicidal behaviors among youth groups.

From the perspective of intervention, identifying suicidal ideation is a crucial first step in preventing suicidal behaviors [17,34]. Meanwhile, research indicates that methods of suicide that are often fatal tend to have a short duration, which limits opportunities for intervention and can lead to death by suicide [25,34,41,42]. As a result, this study emphasizes suicidal ideation identification and suicidal methods as prerequisites for effective intervention. Furthermore, the social-ecological theory suggests that the success of suicide interventions is closely linked to support systems at various levels: societal, community, relational, and individual [43,44,45]. This study selects family, peers, and school support as essential resources for suicide intervention among young adults. A conditional combination model illustrating the pathways for intervening in suicidal behaviors among youth is presented in Figure 2.

In conclusion, most existing studies focus on the net effects of isolated factors, failing to capture the complex, synergistic combinations of conditions that may characterize real-world suicide processes. To fill this gap, this study is based on the above-mentioned adolescent suicide identification and intervention model, and employs qualitative comparative analysis (QCA) method to achieve two main goals: first, to identify the necessary conditions and sufficient configurational pathways for the successful identification of suicidal ideation among Chinese youth; and second, to unravel the complex combinations of conditions associated with successful versus failed interventions in their suicidal behaviors. Based on this work, this research will provide a nuanced, context-rich understanding that can inform more targeted and effective suicide prevention strategies.

## 2. Method

### 2.1. Participants

The University Institutional Academic Ethics Review Committee approved the research protocol, allowing for the investigation of Chinese young people aged 18–29. Written informed consent was obtained from all participants. During the interview process, this study invited professionals to provide necessary psychological support to the participants and allowed them to withdraw midway. Meanwhile, this study only disclosed the parts that participants agreed to make public, and the rest of the information was treated with concealment.

Snowball sampling is a non-probability sampling method that utilizes social networks to access populations that are difficult to reach through conventional recruitment methods [46]. It is suitable for recruiting cases (the specific suicidal incident) and participants (the individuals who were interviewed to provide information about that case) for this study. The process unfolded as follows:

Firstly, this study recruited informants from communities, schools, and hospitals, including community grid workers (n = 5), university counselors (n = 15), mental health center doctors (n = 2), and psychological counselors (n = 2). These informants were chosen for their high likelihood of contact with young people experiencing psychological distress or suicidal behaviors. Secondly, through the networks of these informants, we obtained referrals for 93 potential cases of youth suicide (including both attempted and completed suicide). Thirdly, this study selected the cases who were aged between 18 and 29 years and had a clear history of suicidal behaviors (including ideation with a specific plan, attempt, or completion) within the past two years. Meanwhile, relevant personnel of these cases were willing to participate in the investigation. Finally, 47 cases were deemed eligible and were included in the final study sample.

For each of the 47 included cases, this study aimed to collect data from one to two key participants who had the most direct knowledge of the suicidal incident. In the six cases of completed suicide, the primary participants were parents or close relatives. For the remaining cases of attempted suicide, participants included the young person themselves, supplemented by interviews with parents, relatives, mentors, counselors, or friends to triangulate data. The distribution of cases and their corresponding participants is detailed in Table 1.

### 2.2. Measurements

Data collection utilized semi-structured interviews focusing on four dimensions of youth suicide processes: (1) Basic background: psychological disorders and stressors; (2) Suicidal communication: recipients, methods, and timing of ideation disclosure; (3) Suicide behaviors: suicidal methods, time, and locations; (4) Suicide intervention: support providers, implemented measures, and intervention outcomes. The interviews lasted 30 to 60 min. This study encoded interview data to extract information related to suicide communication, suicide methods, family support, school support, and peer support during suicide. The interview questionnaire can be found in Appendix A.

Based on prior research literature and theoretical frameworks, psychological disorders, suicidal history, suicide communication, suicidal time, and suicidal location are defined as the independent variables of suicidal ideation identification. Meanwhile, suicidal ideation identification, suicidal methods, family support, school support, and peer support are regarded as the independent variables of suicide intervention. Before encoding the interview data, this study defined the core connotations of these 11 key variables: ***(1) Psychological disorders*** are defined as a syndrome characterized by clinically significant disturbance in an individual’s cognition, emotion regulation, or behavior, such as depression, anxiety, bipolar disorder [47]. ***(2) Suicidal history*** refers to the experience of attempting suicide in the past; people with a history of suicide are more likely to receive attention [48]. ***(3) Suicidal communication*** is regarded as a cry for help and can be classified as verbal and non-verbal and also divided into direct and indirect communication [49]. ***(4) Suicide time*** refers to the time when an individual engages in suicidal behavior or attempts suicide, which is divided into daytime (6:00 a.m.–22:00 p.m.) and nighttime (22:00 p.m.–6:00 a.m.) in this article. ***(5) Suicidal location*** refers to the specific address where an individual engages in suicidal behavior or attempts suicide. The main research object of this article is young students, so it is divided into on-campus and off-campus. ***(6) Suicidal ideation identification*** refers to the discovery and prediction of potential suicide risks by analyzing an individual’s behaviors, words, psychological characteristics, and other information. Timely identification of an individual’s suicidal intention is beneficial in stopping the process of suicide [34]. ***(7) Suicidal methods*** refer to the means adopted by an individual when attempting suicide. Research has shown that the mortality rate of methods such as jumping off buildings, hanging, and taking poison is much higher than that of excessive medication and wrist cutting [25]. Therefore, it can be divided into high high-fatal methods and low-fatal methods. ***(8) Family support*** refers to emotional, material, and other support from family members during the suicide process, including parents, siblings, and relatives [50]. ***(9) School support*** refers to emotional, material, and other support from school staff during the suicide process, such as teachers, administrators, medical personnel, security staff, and psychological counselors [51]. ***(10) Peer support*** refers to emotional, material, and other support from peers, including classmates, roommates, and friends [52]. ***(11) Suicide intervention*** refers to a series of strategies, plans, and actions aimed at preventing suicidal behaviors [25]. This study mainly focuses the results of interventions, divided into success (survived) and failure (died).

Based on these definitions, this study established coding rules for each variable, clarified the criteria for assigning a value of 1 or 0, and selected representative excerpts from actual interview transcripts as references for coding. Two experienced PhD researchers were invited to independently code the variables for the cases, with a third researcher serving as an arbiter in case of disagreements. Since the coding was based on objective descriptions of behaviors or outcomes, the inter-coder reliability test yielded a Kappa value of 0.93 (>0.85). Thus, the coding results demonstrate high reproducibility and validity. The coding rules of the variables and coding results are shown in the Supplementary Materials.

### 2.3. Analysis Approaches

This study aims to explore the differences between individuals who attempt suicide and those who complete suicide among Chinese youth. It used the crisp-set qualitative comparative analysis (csQCA) method, which helps examine the various pathways related to identifying suicidal thoughts and intervening in suicidal behaviors. There are several reasons for choosing csQCA as follows:

First, csQCA enables researchers to identify complex interactions and address equifinality, making it a valuable tool for exploring the different pathways for recognizing suicidal ideation and intervening in suicidal behaviors [53,54]. Second, one of the main advantages of csQCA is its ability to compare configurations, which is beneficial for distinguishing differences between suicide attempters and completers [55,56]. Third, youth suicide is a sensitive and stigmatized issue in China, making it challenging to gather a large amount of data. csQCA is particularly well-suited for investigations involving small to medium sample sizes [55].

The fsQCA is a specialized software program developed specifically for conducting Qualitative Comparative Analysis (QCA), which is widely recognized as a standard tool in the field for implementing the core analytical procedures of the csQCA methodology [53]. Therefore, this study used fsQCA 4.1 software to finish the data analysis: (1) Based on empirical and theoretical knowledge, set thresholds of outcome variables and condition variables to construct a binary data table. (2) Use the single-condition necessity analysis to evaluate the subset relationship between outcome and condition variables. (3) Construct the truth table. (4) Perform the configuration analysis to simplify condition combinations. (5) Apply a robustness test to verify the the stability of condition combinations.

## 3. Results

Table 2 shows that 46.8% of cases were male, and 53.2% were female. Their ages ranged from 18 to 28, and 89.4% were younger than 25. From the education level, 70.2% were undergraduate students, and only 29.8% were graduate students. 74.5% lived on campus, and 25.5% lived off campus. There were 37 cases with psychological disorders, accounting for 78.7%. The types of suicidal behaviors, suicidal plan, drug overdose, slitting wrists, jumping from a building, and drowning, respectively, accounted for 42.6%, 29.8%, 6.4%, 17.0%, and 4.2%. From the intervention results, attempted suicide accounted for 87.2%, while suicide deaths accounted for 12.8%.

### 3.1. Paths of Suicidal Ideation Identification

**Necessity Analysis.** Single-condition necessity analysis was conducted to identify conditions that may be essential for successful identification. As shown in Table 3, three conditions—suicidal communication, daytime occurrence, and on-campus location—demonstrated high consistency scores (0.9) with the outcome, meeting the conventional threshold for potential necessity [53]. However, a nuanced interpretation is warranted by their coverage scores. Suicidal communication exhibited both high consistency and substantial coverage (0.818), suggesting it is a central and broadly relevant condition for identification. In contrast, daytime occurrence and on-campus location showed only moderate coverage (approximately 0.5), indicating they are context-dependent necessities. This pattern suggests that while these temporal and spatial conditions are highly prevalent in successful identification scenarios, a significant portion of cases are successfully identified even in their absence (e.g., at night or off-campus).

**Figure 3:**
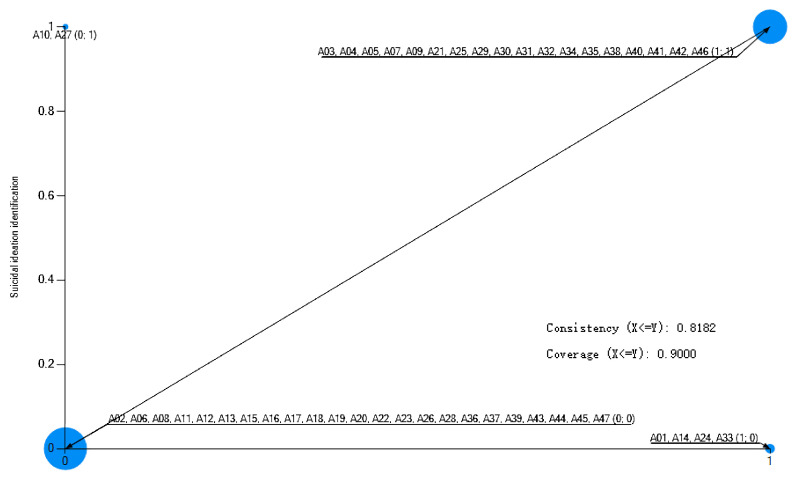
A scatter plot of suicide communication and suicidal ideation identification. Note: Cases in the upper-left quadrant represent successful identification despite the absence of the condition, illustrating boundary scenarios.

**Figure 4:**
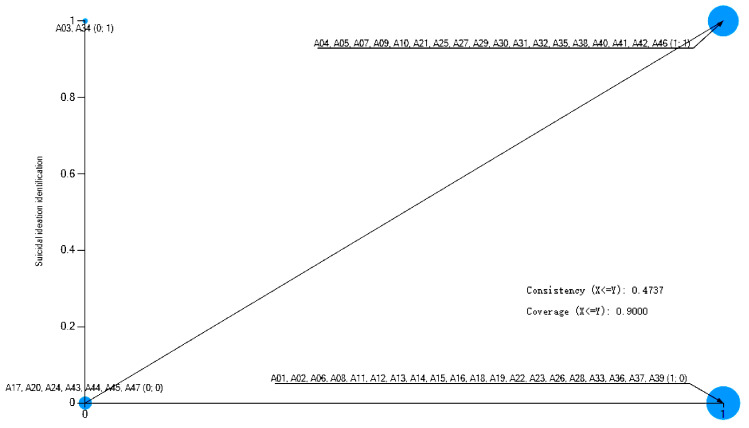
A scatter plot of suicide time and suicidal ideation identification. Note: Cases in the upper-left quadrant represent successful identification despite the absence of the condition, illustrating boundary scenarios.

**Figure 5:**
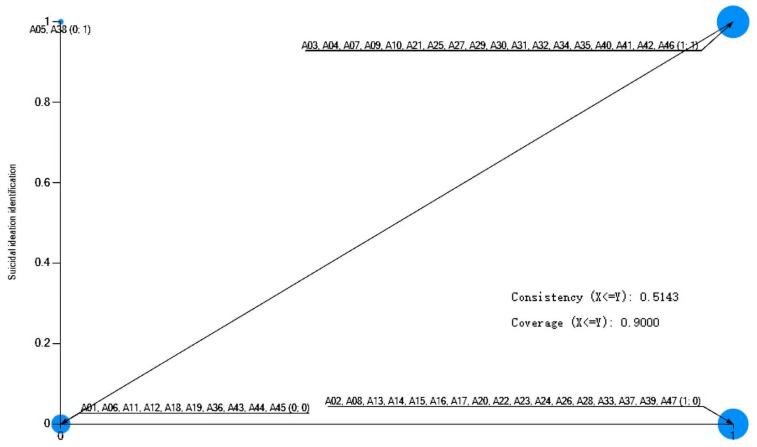
A scatter plot of suicide location and suicidal ideation identification. Note: Cases in the upper-left quadrant represent successful identification despite the absence of the condition, illustrating boundary scenarios.

These boundary cases are visually apparent as outliers in the upper-left quadrant of the scatterplots (Figure 3, Figure 4 and Figure 5), where successful identification (Y = 1) occurs despite the absence of the condition (X = 0). The existence of these cases reveals the limits of models relying solely on physical and temporal context. They likely represent scenarios where identification is achieved through alternative mechanisms, such as night patrol of the school (such as Case A10 without suicidal communication), proactive online monitoring (such as Case A27 in the nighttime), and weekly student statistics (such as Case A38 off campus), which can transcend traditional spatial and temporal boundaries. Therefore, while suicidal communication, daytime, and on-campus location are robust facilitative contexts, their necessity is not absolute and can be supplemented by other systematic identification efforts.

**Truth Table Analysis.** The truth table should have two different combinations of criteria. Given the small sample size and the binary data variables, this study set a frequency threshold of 2, a consistency threshold of 0.8. The results of the truth table analysis are shown in Table 4. There are 3 condition combinations of successful identification and 9 condition combinations of failed identification.

**Sufficiency Analysis.** The csQCA configuration analysis generates complex, intermediate, and parsimonious solutions by eliminating situations that do not meet the frequency and consistency thresholds. In a frequency threshold of 2, a consistency threshold of 0.8, and a PRI threshold of 0.8, the software generated only a single, unique intermediate solution and did not report any logically equivalent solutions. As shown in Table 5, the intermediate solution for the successful identification of suicidal ideation yielded two configurations. These collectively accounted for 35% of positive cases with high consistency (overall solution consistency = 1), all exceeding the adequacy threshold (>0.75). The row coverages of Path 1 and Path 2 were 20% and 15%. Path 1 had the highest unique coverage (20%), which means that Path 1 makes a contribution to explaining the successful identification of suicidal ideation.

For the failed identification of suicidal ideation, the intermediate solution identified four configurations, explaining 66.7% of negative cases with high consistency (overall solution consistency = 1). The row coverages of Path 3, Path 4, Path 5, and Path 6 were 14.8%, 14.8%, 18.5%, and 33.3%, respectively. Path 6 had the highest unique coverage (25.9%), which means that Path 6 makes a contribution to explaining the failed identification of suicidal ideation. Both solutions met QCA validity standards, revealing asymmetric causal patterns between success and failure pathways [53].

**Sensitivity Test.** To assess the robustness, this study conducted a sensitivity analysis by decreasing the frequency threshold from 2 to 1. The software generated only a single, unique intermediate solution and did not report any logically equivalent solutions. A comparison between the primary analysis (Table 5) and the sensitivity analysis (Table 6) reveals that the core configuration paths for both successful and failed identification of suicidal ideation remained relatively stable. Meanwhile, the overall coverage of the model was significantly improved after relaxing the standards.

From the successful identification, the core conditions present in Paths 1 and 2 were preserved in the sensitivity analysis (Paths 1′ and 2′), underscoring their stability. Table 7 presents an additional pathway (Path 3′), increasing the overall solution coverage to 89.5%. This suggests that Path 3′ represents a common identification scenario in practice, while this pathway did not remain stable. Through the analysis of cases with conflicting outcomes, this study found that the instability of Path 3 reveals the inherent heterogeneity of suicide communication. There is a certain spatiotemporal distance in online communication, which may lead to lower effectiveness in identifying suicide signals compared to offline interactions (including language, behavior, demeanor, etc.).

On the other hand, the number of paths for failed identification increased from 3 in the primary analysis to 4, and the overall coverage increased from 66.7% to 81.5%. Specifically, the newly emerged Path 4′ achieved an increase in coverage by merging Path 3 and Path 4 from the primary analysis. However, this process masked the heterogeneity of key variables such as ‘psychological disorders’ and ‘suicidal location’, leading to the emergence of contradictory cases and reducing the stability and theoretical clarity of this path. Furthermore, the evolution from Path 5 to Path 5′ demonstrates that the ‘absence of suicidal communication’ serves as a more stable core condition than ‘psychological disorders’. The comparison between Path 6 and Path 6′ indicates that the ‘absence of suicidal communication’ is a more explanatory and stable core condition than ‘being off-campus’.

### 3.2. Paths of Suicide Behaviors Intervention

**Figure 6:**
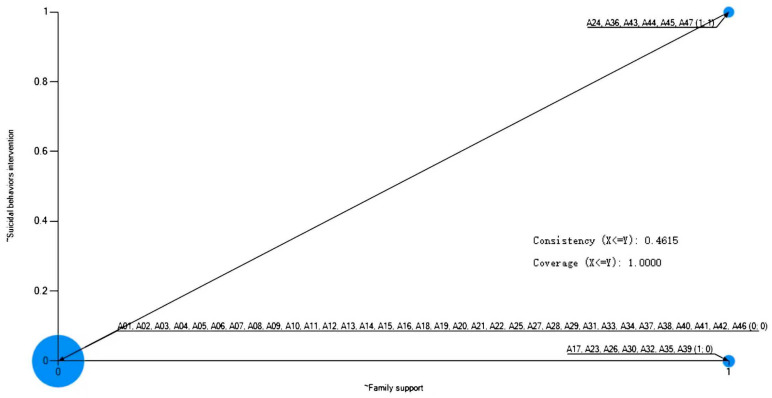
A scatter plot of family support and suicidal behaviors intervention.

**Necessity Analysis.** The analysis of necessary conditions for intervention failure yielded one clear finding: the absence of family support (~family support) is a necessary condition for failed intervention, demonstrating perfect consistency (Table 7). As visually confirmed in the scatterplot (Figure 6), all cases of intervention failure cluster in the region where family support is absent (right side of the plot). No failure cases occurred when family support was present. The coverage of this condition (0.462) indicates that it explains nearly half of the instances of intervention failure.

This finding reveals a critical asymmetric relationship. The consistent absence of family support across all failure cases underscores its role as a critical barrier; when it is missing, the intervention system possesses a fundamental vulnerability that can lead to tragic outcomes. However, it is crucial to emphasize that the presence of family support does not, by itself, guarantee successful intervention. The moderate coverage value confirms that other pathways to failure exist even when family support is present, such as failures in suicide ideation identification (such as Case A24), the use of highly lethal means (such as Case A45), or a concurrent lack of school and peer support (such as Case A36). Thus, family support functions as a necessary but insufficient protective factor; its absence dramatically increases risk, but its presence must be complemented by other supportive conditions to ensure intervention success.

**Truth Table Analysis.** The truth table should have two different combinations of criteria. Given the small sample size and the binary data variables, this study set a frequency threshold of 2, a consistency threshold of 0.8, and a PRI threshold of 0.8. The results of the truth table analysis are shown in Table 8. There are 8 condition combinations of successful suicide intervention and 1 condition combinations of failed suicide intervention.

**Sufficiency Analysis.** The csQCA configuration analysis generates complex, intermediate, and parsimonious solutions by eliminating situations that do not meet the frequency and consistency thresholds. In a frequency threshold of 2, a consistency threshold of 0.8, and a PRI threshold of 0.8, the software generated only a single, unique intermediate solution and did not report any logically equivalent solutions.

As shown in Table 9, the intermediate solution for the successful intervention of suicidal behaviors yielded five causal configurations. These collectively accounted for 92.7% of positive cases with high consistency (overall solution consistency = 1.00), all exceeding the adequacy threshold (>0.75). The row coverage of Path 7, Path 8, Path 9, Path 10, and Path 11 was 58.5%, 31.7%, 29.3%, 19.5%, and 24.4%, respectively. Path 7 had the highest unique coverage (17.1%), which means that Path 7 makes a contribution to explaining the successful identification of suicidal ideation. For the failed intervention of youth suicide, the intermediate solution identified two configurations, explaining 66.7% of negative cases with perfect consistency (overall solution consistency = 1.00). The row coverage and unique coverage were 66.7%. All solutions met QCA validity standards, revealing asymmetric causal patterns between success and failure pathways [53].

**Sensitivity Test.** To assess the robustness, this study conducted a sensitivity analysis by increasing the frequency threshold from 2 to 1. The software generated only a single, unique intermediate solution and did not report any logically equivalent solutions. A comparison between the primary analysis (Table 9) and the sensitivity analysis (Table 10) reveals that the core configuration paths for both successful and failed interventions of suicidal behaviors remained relatively stable. Meanwhile, the overall coverage of the model was significantly improved after relaxing the standards.

The results of both tables indicate that school intervention plays a central role in the successful intervention pathway. The number and coverage of successful intervention paths were similar in the two models, but there were slight differences in the configuration of conditions. Compared to the results of the sensitivity analysis, Path 7 enhances the coverage and stability of successful home-school collaborative interventions by adding the core precondition for non-lethal suicide methods. Path 8 and Path 10, respectively, strengthen intervention power to increase the probability of success by incorporating family support and peer support, and Path 9 and Path 11 reinforce suicide prevention through the assistance of ‘suicide identification’.

For the failed intervention, by integrating the conditional structures of Path 12′ and Path 13′, Path 12 achieves significantly enhanced stability at the cost of reduced coverage, thereby effectively avoiding more contradictory cases. For instance, in some cases where individuals attempt suicide by jumping from a height, they may firmly reject help and support from family and teachers, ultimately being persuaded to abandon the act solely through peer intervention. Such cases partially challenge the explanatory power of Path 12′ and Path 13′. The critical insight provided by Path 12 is that successful suicide often requires the simultaneous fulfillment of two conditions: evading the attention of effective social support networks and employing determined suicidal means.

## 4. Discussion

In conclusion, under strict criteria of a frequency threshold of 2, a consistency threshold of 0.8, and a PRI consistency threshold of 0.8, the suicide identification and suicide intervention pathways derived from 47 cases (Path 1 to Path 12 in the main analysis) demonstrated good robustness. It is important to note that the findings from this csQCA study, based on binary coding and a specific sample of Chinese youth, are primarily hypothesis-generating. The configurational paths this study identified represent robust associations within the data but should not be interpreted as definitive causal relationships. They provide valuable, nuanced insights that can guide future research with larger, more diverse samples and longitudinal designs to test these potential pathways.

### 4.1. Paths of Suicidal Ideation Identification

Results indicate the effective communication of suicidal intent is a core condition for successful identification of suicidal ideation among young people, while “silence” is a common characteristic across all failure pathways. Suicidal communication serves as a critical distress signal, with approximately half of those who die by suicide having expressed their intentions to people around them [49]. However, due to factors such as the gender of the individual, communication style, and differences in recipients’ awareness, these signals may not be recognized promptly or accurately [38,57]. As digital natives, adolescents rely heavily on online environments for social interaction, emotional expression, and identity formation. Therefore, enhancing online suicide monitoring and suicide risk early-warning systems represents an essential approach to improving suicide identification rates [58].

Specifically, there are two effective paths for identifying suicidal ideation among Chinese youth. Path 1 suggests that impulsive suicide attempters are likely to express strong suicidal intentions with others during the daytime, making it crucial to pay attention to these signals. Previous studies have shown that impulsive attempters often have a higher degree of self-rescue ideation and are more likely to threaten or express anger toward others, which can serve as a cry for help [49,59]. Therefore, accurately interpreting the words and behaviors of impulsive suicide victims can be key to effectively identifying suicidal ideation. Path 2 suggests that young people with psychological disorders are more easily identified as having suicidal thoughts when they express warning signs to others on campus, particularly during the day. Research has shown that the frequency of these suicide warning signs is higher in individuals with psychological disorders compared to other groups [60]. Additionally, when these signals occur during the day or in public places, the likelihood of others recognizing their suicidal ideation increases [39,40]. Therefore, implementing the Campus Gatekeeper Program is essential for preventing suicide among young people with mental health issues.

On the other hand, this study identifies two significant issues in failed suicide prevention cases. Path 4 indicates that schools have inadequate prevention efforts for teenagers who do not exhibit a history of suicidal behavior or communicate suicidal thoughts. Some studies demonstrate that 19.4% of young people show no warning signs of suicide [61], and nearly 80% die following their first suicide attempt [62]. Collectively, these findings indicate a lack of proactive mechanisms for recognizing suicidal behavior and an absence of universal suicide prevention education. Paths 3, 5, and 6 reveal that inadequate suicide prevention mechanisms exist for young people with psychiatric disorders, particularly in terms of support from off-campus environments. Research has shown that nearly 50% of young suicide attempters do not receive formal psychological treatment. This is primarily due to parents underestimating the risk of suicide and the community’s lack of support for high-risk youth groups [63,64]. Additionally, 55.6% of young people who commit suicide do so at home [61], which means the risk of suicide outside of school is higher. Therefore, it is crucial to strengthen the attention and support that families and communities provide to high-risk youth groups in order to prevent suicide.

### 4.2. Paths of Suicidal Intervention

Results showed that there are five successful paths to realizing suicidal intervention in Chinese young people. Paths 7–10 collectively highlight that the presence of school support is a central component associated with successful intervention. This finding is aligned with the theoretical emphasis on school as a critical intervention setting, including gatekeeper training programs, mandatory referral mechanisms, professional psychological interventions, emergency first aid, and campus patrols, which have effectively reduced the risk of student suicides [65,66,67].

Specifically, Path 9 indicates that school support can play an independent role in suicide intervention when individuals’ suicidal intentions are successfully identified and they engage in non-fatal suicidal behaviors (accounting for 29.3%). This is largely due to the fact that non-fatal suicidal behaviors occur on-campus (such as self-injury or attempted suicide) usually have a longer intervention window, and schools can reduce the recurrence rate through psychological first aid and follow-up psychological interventions [51,68,69]. It can be seen that effective suicide identification and non-fatal suicide methods are prerequisites, and professional support from schools is the core condition.

It should be pointed out that the collaborative intervention of multiple forces is also an important condition for youth suicide intervention. The synergistic effect of schools and families (Paths 7 and 8), schools and peers (Path 10), and families and peers (Path 11) can compensate for the lack of early suicide identification, and even block fatal suicide behaviors, effectively reducing the risk of youth suicide. Previous studies have also provided similar evidence that youth receiving multidimensional support exhibit markedly lower suicide risks than those relying on singular support sources [70,71]. In addition, family and school support are more important in suicide interventions for high-risk individuals [72,73], which explains 90.2% of the successful cases. Therefore, establishing a collaborative intervention mechanism among schools, families, and peers is effective in reducing youth suicide rates.

On the other hand, the results found one path of failed suicidal intervention. Path 12 indicates that the dual lack of timing and intervention power in suicide intervention is a key factor leading to the failure of youth suicide intervention. Fatal suicidal behaviors often exhibit a brief intervention window, such as jumping from a building, drowning, and shooting, which limits the opportunities for suicide intervention [34,42,72]. In this study, the cases of suicide were all high-risk groups who chose to avoid people around them and resorted to fatal suicide methods in the nighttime, so that no one from school, family, or peers noticed or intervened. Therefore, limiting fatal suicide methods is crucial, such as closing rooftop passages, strengthening lakeside guardrails, increasing monitoring of hazardous areas, and limiting the amount of medication [17,34].

Some shortcomings of this study need to be noted. Firstly, due to the sensitivity and specificity of youth suicide issues, this study collected a small number of cases. It may not be able to further explain the differences among different groups, such as gender, economic level, sexual orientation, living environment, etc. Secondly, although the indicators used in this study were objective, they may still be affected by the recall bias of the respondents, thereby affecting the accuracy of the research results. These limitations indicate future research possibilities. Thirdly, this study constructed a binary data table based on the results of case interviews without using structured questionnaires for measurement, which may limit the depth and validity of data analysis. Fourth, this study combined suicide decedents with attempt survivors, which represent generalized patterns across a heterogeneous group and should not be viewed as outcome-specific. These shortcomings constitute the direction for future research.

## 5. Conclusions

Despite its limitations, this study contributes to the understanding of suicide identification and intervention among young people. Continuous supervision of individuals with psychological disorders or a history of suicide is crucial for early detection, and multi-group participation is key to successful intervention, especially school and family support. This study also found three main issues: a lack of proactive identification of individuals without warning signals, insufficient attention from families and communities to young people with psychological disorders, and inadequate physical limitations on the fatal suicidal behaviors. Therefore, this study highlights the importance of strengthening continuous attention to suicide signals among high-risk youth groups, limiting lethal suicide methods, promoting network monitoring and suicide risk warning, increasing psychological services in the community, and improving the collaborative synergy of peers, families, and schools. In future research, we will use structured interviews to collect more samples and investigate suicide identification and intervention mechanisms among different groups, in order to obtain more targeted and inclusive prevention strategies.

## Figures and Tables

**Table 1:** Participants of interviews.

Case Number	Participants	Interview Method
1, 4, 5, 9, 15, 16, 19, 23, 30, 34, 36, 37, 39, 42, 43, 44, 47	Suicide victim and school counselor	Offline interview
8, 13, 18, 28, 40, 46	Suicide victim and mentor/psychological counselor	Offline interview
3, 6, 10, 21, 24, 27, 33	Suicide victim and classmates/roommates	Offline interview
7, 11, 17, 20, 22, 29, 32, 35, 41	Suicide victim and parents/relatives	Online interview
2, 14, 26, 31, 38, 43	Parents and relatives	Online interview
12, 25, 45	Suicide victim and friends	Online interview

**Table 2:** Characteristics of Cases (n = 47).

Variables		N	%
Gender	Male	22	46.80%
	Female	25	53.20%
Age (18–28)	≤24	42	89.40%
	>24	5	10.60%
Education	Undergraduate students	33	70.20%
	Graduate students	14	29.80%
Living locations	On campus	35	74.50%
	Off campus	12	25.50%
Psychological disorders	Yes	37	78.70%
	No	10	21.3%%
Suicidal behaviors	Suicidal plan	20	42.6%
	Overdose	14	29.8%
	Slitting	3	6.4%
	Jumping	8	17.0%
	Drowning	2	4.2%
Suicidal outcome	Survived	41	87.2%
	Died	6	12.8%

**Table 3:** The single-condition necessity analysis of suicidal ideation identification.

	Successful Identification		Failed Identification	
Conditions	Consistency	Coverage	Consistency	Coverage
Psychological disorders	0.800000	0.432432	0.777778	0.567568
~ Psychological disorders	0.200000	0.400000	0.222222	0.600000
Suicidal history	0.300000	0.545455	0.185185	0.454545
~ Suicidal history	0.700000	0.388889	0.814815	0.611111
Suicide communication	0.900000	0.818182	0.148148	0.181818
~ Suicide communication	0.100000	0.080000	0.851852	0.920000
Suicidal time	0.900000	0.473684	0.740741	0.526316
~ Suicidal time	0.100000	0.222222	0.259259	0.777778
Suicidal location	0.900000	0.514286	0.629630	0.485714
~ Suicidal location	0.100000	0.166667	0.370370	0.833333

Note: ‘~’ indicates logical negation and means without.

**Table 4:** Conditional combination truth table of suicidal ideation identification.

Psychological Disorders	Suicidal History	Suicide Communication	Suicidal Time	Suicidal Location	Identification	n	Raw Consist.	PRl Consist.
1	1	1	1	1	1	3	1	1
0	0	1	1	0	1	2	1	1
0	0	1	1	1	1	2	1	1
1	0	1	1	1	0	11	0.8181812	0.8181812
1	1	0	1	1	0	4	0.5	0.5
1	0	1	0	1	0	2	0.5	0.5
1	0	0	1	1	0	7	0	0
1	1	0	1	0	0	3	0	0
1	0	0	0	0	0	2	0	0
1	0	0	1	0	0	2	0	0
0	0	0	0	1	0	2	0	0
0	0	0	1	1	0	2	0	0

Note: The analysis employed crisp-set Qualitative Comparative Analysis (csQCA). The applied thresholds were as follows: frequency threshold = 2; consistency threshold = 0.8. PRI threshold = 0.8.

**Table 5:** Intermediate solution in explaining paths of suicidal ideation identification.

	Successful Identification		Failed Identification			
Conditions	Path 1	Path 2	Path 3	Path 4	Path 5	Path 6
Psychological disorders	⊗ **	⊙ ***	⊙	⊘	⊙	⊙
Suicidal history	⊘ ****	*****	⊗	⊗		⊗
Suicide communication	● *	●	⊗	⊗	⊗	⊗
Suicidal time	⊙	●			⊙	⊙
Suicidal location		●	⊗	⊙	⊗	
Row coverage	0.2	0.15	0.148148	0.148148	0.185185	0.333333
Unique coverage	0.2	0.15	0.074074	0.148148	0.111111	0.259259
Consistency	1	1	1	1	1	1
PRI Consistency	1	0.857414	1	1	0.818182	1
Overall solution consistency	1		1			
Overall solution coverage	0.35		0.666667			

Note: 1. *: ‘●’ indicates the presence of a core condition. **: ‘⊗’ indicates the absence of a core condition. ***: ‘⊙’ indicates the presence of a peripheral condition. ****: ‘⊘’ indicates the absence of a peripheral condition. *****: ‘ ’ indicates that the condition does not matter. 2. The analysis employed crisp-set Qualitative Comparative Analysis (csQCA). The applied thresholds were as follows: frequency threshold = 2; consistency threshold = 0.80; PRI threshold = 0.80.

**Table 6:** The results of sensitivity test.

	Successful Identification			Failed Identification		
Conditions	Path 1′	Path 2′	Path 3′	Path 4′	Path 5′	Path 6′
Psychological disorders	⊗ **	⊙	⊙ ***		●	●
Suicidal history	⊘ ****	*****	● *	⊗		⊘
Suicide communication	●	●	⊙	⊗	⊘	
Suicidal time	⊙	●			⊙	⊙
Suicidal location		●	⊙		⊗	⊗
Row coverage	0.2	0.55	0.2	0.666667	0.185185	0.111111
Unique coverage	0.1	0.45	0.2	0.592593	0.111111	0.037037
Consistency	1	0.846154	1	1	1	1
PRI Consistency	1	1	1	1	1	1
Overall solution consistency	0.850000			1		
Overall solution coverage	0.894737			0.814815		

Note: 1. *: ‘●’ indicates the presence of a core condition. **: ‘⊗’ indicates the absence of a core condition. ***: ‘⊙’ indicates the presence of a peripheral condition. ****: ‘⊘’ indicates the absence of a peripheral condition. *****: ‘ ’ indicates that the condition does not matter. 2. The analysis employed crisp-set Qualitative Comparative Analysis (csQCA). The applied thresholds were as follows: frequency threshold = 2; consistency threshold = 0.80; PRI threshold = 0.80.

**Table 7:** The single-condition necessity analysis of suicidal behaviors intervention.

	Successful Intervention		Failed Intervention	
Conditions	Consistency	Coverage	Consistency	Coverage
Suicidal ideation identification	0.463415	0.950000	0.166667	0.050000
~ Suicidal ideation identification	0.536585	0.814815	0.833333	0.185185
Suicidal methods	0.146341	0.545455	0.833333	0.454545
Peer support	0.585366	0.857143	0.666667	0.142857
~ Peer support	0.414634	0.894737	0.333333	0.105263
School support	0.853659	0.972222	0.166667	0.027778
~ School support	0.146341	0.545455	0.833333	0.454545
Family support	0.829268	1.000000	0.000000	0.000000
~ Family support	0.170732	0.538462	1.000000	0.461538

Note: ‘~’ indicates logical negation and means without.

**Table 8:** Conditional combination truth table of suicidal behaviors intervention.

Suicidal Ideation Identification	Suicide Methods	Peer Support	School Support	Family Support	Intervention	n	Raw Consist.	PRl Consist.
1	0	0	1	1	1	9	1	1
1	0	1	1	1	1	7	1	1
0	0	1	1	1	1	6	1	1
0	0	1	0	1	1	4	1	1
1	0	0	1	0	1	3	1	1
0	1	1	1	1	1	3	1	1
0	0	1	1	0	1	2	1	1
0	0	0	1	1	1	2	1	1
0	1	0	1	1	1	2	1	1
0	1	0	0	0	0	4	0	0

Note: The analysis employed crisp-set Qualitative Comparative Analysis (csQCA). The applied thresholds were as follows: frequency threshold = 2; consistency threshold = 0.8. PRI threshold = 0.8.

**Table 9:** Intermediate solutions in explaining paths of suicidal behaviors intervention.

	Successful Intervention					Failed Intervention
Conditions	Path 7	Path 8	Path 9	Path 10	Path 11	Path 12
Suicidal ideation identification	*****	⊘ ****	⊙ ***	⊘	⊘	⊘
Suicidal methods	⊗ **		⊗	⊗	⊗	●
Peer support			⊘	⊙	⊙	⊘
School support	● *	●	●	●		⊘
Family support	⊙	⊙			⊙	⊗
Row coverage	0.585366	0.317073	0.292683	0.195122	0.243902	0.666667
Unique coverage	0.170732	0.121951	0.0731707	0.0487805	0.097561	0.666667
Consistency	1	1	1	1	1	1
PRI Consistency	1	1	1	1	1	1
Overall solution consistency	1.000000					1.000000
Overall solution coverage	0.926829					0.666667

Note: 1. *: ‘●’ indicates the presence of a core condition. **: ‘⊗’ indicates the absence of a core condition. ***: ‘⊙’ indicates the presence of a peripheral condition. ****: ‘⊘’ indicates the absence of a peripheral condition. *****: ‘ ’ indicates that the condition does not matter. 2. The analysis employed crisp-set Qualitative Comparative Analysis (csQCA). The applied thresholds were as follows: frequency threshold = 1; consistency threshold = 0.80; PRI threshold = 0.80.

**Table 10:** The results of robustness test.

	Successful Intervention					Failed Intervention	
Conditions	Path 7′	Path 8′	Path 9′	Path 10′	Path 11′	Path 12′	Path 13′
Suicidal ideation identification	⊘	⊘ ****	*****	⊘		⊘	⊘
Suicidal methods			⊘	⊘	⊘	⊙ ***	
Peer support		⊘	⊘		⊙		⊙
School support	●	●	● *	●		⊗ **	⊗
Family support	●				●	⊗	⊗
Row coverage	0.317073	0.146341	0.365854	0.268293	0.439024	0.833333	0.833333
Unique coverage	0.0731707	0.0243902	0.292683	0.0487805	0.292683	0.166667	0.166667
Consistency	1	1	1	1	1	1	1
PRI Consistency	1	1	1	0.916667	1	1	1
Overall solution consistency	1.000000					1.000000	
Overall solution coverage	1.000000					1.000000	

Note: 1. *: ‘●’ indicates the presence of a core condition. **: ‘⊗’ indicates the absence of a core condition. ***: ‘⊙’ indicates the presence of a peripheral condition. ****: ‘⊘’ indicates the absence of a peripheral condition. *****: ‘ ’ indicates that the condition does not matter. 2. The analysis employed crisp-set Qualitative Comparative Analysis (csQCA). The applied thresholds were as follows: frequency threshold = 1; consistency threshold = 0.8; PRI threshold = 0.8.

## Data Availability

The original data presented in the study are openly available in Science Data Bank at https://doi.org/10.57760/sciencedb.22438.

## Supplementary Materials

The following supporting information can be downloaded at: https://www.mdpi.com/article/10.3390/healthcare13233128/s1, Table S1. Code-rule; Table S2. coding-results.

## Appendix A. The Interview Questionnaire

1. What is the basic situation of XX? (including gender, age, educational level, school, place of residence, family background, personality, etc.)
2. Have XX been diagnosed with mental illness before committing suicide? Have you ever attempted suicide?
3. What caused XX to have suicidal thoughts (most recently)?
4. Did XX at the time mention or imply to others that they had suicidal thoughts? Did XX leave a suicide note?
5. When was the time when XX attempted suicide? Where?
6. What suicide methods did XX plan or have already adopted at that time?
7. Who discovered at that time that XX wanted to commit suicide or carried out suicide? How to discover?
8. Who provided assistance after discovering XX suicide? What measures have been taken to intervene?
9. After intervention, what is the status of XX?
