# Supplementary material for: Paths of Suicidal Ideation Identification and Suicidal Behavior Intervention: A Qualitative Comparative Analysis of Chinese Young People
Source: Healthcare (Basel). 2025 Dec 1;13(23):3128. doi: 10.3390/healthcare13233128 (PMC12692109; doi:10.3390/healthcare13233128)
Supplement: Supplementary file 1 [file healthcare-13-03128-s001.zip › Table S1. Code-rule.pdf]

Code-rule

Variables	Definition of variables	Coding rules of variables and coding examples	
		Coded as 1	Coded as 0
Psychological disorders	Psychological disorders are defined as a syndrome characterized by clinically significant disturbance in an individual’s cognition, emotion regulation, or behavior, such as depression, anxiety, bipolar disorder[47].	Coding rule: The interviewee described that the case had a certain psychological disorder or was receiving psychological treatment.<br>Example: 'I was diagnosed with severe depression in high school and has been taking medication and treatment ever since.'	Coding rule: The interviewee clearly states that the case has no mental illness or has not undergone psychological treatment.<br>Example: 'My daughter was just impulsive and went to a professional institution for diagnosis after the incident, and she didn’t get any psychological problems.'
Suicidal history	Suicidal history refers to the experience of attempting suicide in the past; people with a history of suicide are more likely to receive attention[48]	Coding rule: The interviewee mentioned that the case had experienced attempted suicide, including suicidal thoughts and actions.<br>Example:'It’s the third attempt at suicide this year, and we have been paying close attention to him.'	Coding rule: The interviewee mentioned in the case that this was their first attempt at suicide.<br>Example: 'The pressure of this exam is too high, and I feel like I can't survive for the first time.'
Suicide communication	Suicidal communication is regarded as a cry for help and can be classified as verbal and non-verbal and also divided into direct and indirect communication[49]	Coding rule: The interviewee described the abnormal words or behaviors of the case before suicide<br>Example: 'I posted 'Goodbye, world' on my social media in QQ, and my friend saw it and immediately called the teacher over.'	Coding rule: The interviewee clearly stated that there were no abnormal behaviors or any suicide signals detected in the case prior to suicide<br>Example:'We checked her phone and there were no signs of suicide.'
Suicidal time	Suicide time refers to the time when an individual engages in suicidal behavior or attempts suicide, which is divided into daytime (6:00 am-22:00pm) and nighttime (22:00pm-6:00 am) in this article.	Coding rule: The interviewee described that the suicide attempt occurred during the daytime(6:00-22:00).<br>Example: 'The security guard noticed her standing on the rooftop of the teaching building during a noon patrol, losing control of her emotions.'	Coding rule: The interviewee described that the suicide attempt occurred during the nighttime(22:00-6:00).<br>Example: 'After checking the surveillance footage, it was found that he jumped off the building at 4:00 am.'
Suicidal location	Suicidal location refers to the specific address where an individual engages in suicidal behavior or attempts suicide. The main research object of this article is young students, so it is divided into on-campus and off-campus.	Coding rule: The interviewer described that the suicide attempt occurred on campus<br>Example: 'I took 2.5g of Quetiapine and was lying in the dormitory waiting to die.'	Coding rule: The interviewer described that the suicide attempt occurred off campus<br>Example: 'She secretly took a fruit knife and cut her wrist while I was cooking in the kitchen.'

Suicidal ideation identification	Suicidal ideation identification refers to the discovery and prediction of potential suicide risks by analyzing an individual's behaviors, words, psychological characteristics, and other information[34].	Coding rule: The interviewee clearly described someone discovering the suicide signals before the event, including verbal or behavioral cues.<br>Example: 'At that time, his roommate saw him buy a knife and immediately reported it to me.'	Coding rule: The interviewee stated that no one noticed or missed the suicide signal before the event.<br>Example: 'He sent me a message at 3:00 am, and I only saw it at 6:00 am, which is already too late.'
Suicidal methods	Suicidal methods refer to the means adopted by an individual when attempting suicide.[25].	Coding rule: The interviewee described the case as committing suicide through fatal behaviors such as jumping from a building, drowning, and taking poison[25].<br>Example: 'I didn't expect him to choose the most decisive way to commit suicide by jumping off a building.'	Coding rules: The interviewee described that the case involved non-fatal behaviors such as wrist cutting, self harm, and excessive medication for suicide.<br>Example: 'I thought taking medication was the least painful way to commit suicide, but it was too painful when it worked.'
Family support	Family support refers to emotional, material, and other support from family members during the suicide process, including parents, siblings, and relatives [50].	Coding rule: The interviewee described that the family actively intervened during the suicide process, such as comforting, sending to the hospital, and seeking help.<br>Example: 'It was his mother who found that he had eaten all the medicine and immediately sent him to the hospital for rescue.'	Coding rule: The interviewee described that the family did not or could not provide support during the suicide process.<br>Example: 'After the incident, we invited his parents to school, but they refused.'
School support	School support refers to emotional, material, and other support from school staff during the suicide process, such as teachers, administrators, medical personnel, security staff, and psychological counselors	Coding rule: The interviewee described that the school actively intervened during the suicide process, such as comforting, sending to the hospital, ,and seeking help.<br>Example:'After the counselor discovered it, he immediately took me to the hospital for emergency treatment and stayed by my side.'	Coding rule: The interviewee described that the school did not or could not provide support during the suicide process.<br>Example: 'He has taken a leave of absence from school and committed suicide multiple times at home, but his mother has consistently refused the school's support.'
Peer support	Peer support refers to emotional, material, and other support from peers, including classmates, roommates, and friends[52].	Coding rule: The interviewee described that friends/classmates actively intervened during the suicide process, such as comforting, sending to the hospital, and seeking help.<br>Example: 'My friend was always talking to me beside me at that time, distracting my attention and comforting me.'	Coding rule: The interviewee describes that no friends or classmates intervened in the suicide process.<br>Example: 'She often argues with her roommates, and on the day of her suicide, no one was in the dormitory.'
Suicide intervention outcome	Suicide intervention refers to a series of strategies, plans, and actions aimed at preventing suicidal behaviors[25].	Coding rule: The interviewee clearly described that the suicide process was terminated and the case survived.<br>Example: 'While cutting my wrist, my boyfriend immediately snatched the knife away.'	Coding rule: The interviewee described that the case had died by suicide.<br>Example: 'The next morning, someone found her drowning in Jincheng Lake.'
